# Effects of Exercise Interventions on Weight, Body Mass Index, Lean Body Mass and Accumulated Visceral Fat in Overweight and Obese Individuals: A Systematic Review and Meta-Analysis of Randomized Controlled Trials

**DOI:** 10.3390/ijerph18052635

**Published:** 2021-03-05

**Authors:** Hyun Suk Lee, Junga Lee

**Affiliations:** 1 Graduate School of Education, Chung-Ang University, Seoul 06974, Korea; hslee@cau.ac.kr; 2 Sports Medicine and Science, Global Campus, Kyung Hee University, Gyeonggi-do 17104, Korea

**Keywords:** fat, obesity, randomized controlled trials, meta-analysis

## Abstract

(1) Background: Exercise interventions for overweight and obese individuals help reduce accumulated visceral fat, which is an indicator of cardiometabolic risk, but the effectiveness of these interventions is controversial. The purpose of this meta-analysis was to investigate the effectiveness of exercise interventions in overweight and obese individuals in order to reduce weight, body mass index (BMI), and accumulated visceral fat, and increase lean body mass. (2) Methods: Databases were used to select eligible studies for this meta-analysis. Randomized controlled trials with control and experimental groups were included. The degrees of effectiveness of the exercise interventions were computed to assess the benefits on reducing weight, BMI, and accumulated visceral fat, and increasing lean body mass. (3) Results: Sixteen studies were included in this meta-analysis. Participation in exercise interventions reduced weight (*d* = −0.58 (95% confidence interval (CI), −0.84–−0.31; *p* < 0.001; *k* = 9)), BMI (*d* = −0.50 (95% CI, −0.78–−0.21; *p* < 0.001; *k* = 7)), and accumulated visceral fat (d = −1.08 (95% CI, −1.60–−0.57; *p* < 0.001; *k* = 5)), but did not significantly increase lean body mass (*d* = 0.26 (95% CI, −0.11–0.63; *p* = 0.17; *k* = 6)). The average exercise intervention for overweight and obese individuals was of moderate to vigorous intensity, 4 times per week, 50 min per session, and 22 weeks duration. (4) Conclusions: Participating in exercise interventions has favorable effects on weight, BMI, and accumulated visceral fat. Further studies considering different modalities, intensities, durations, and measurements of fatness need to be conducted.

## 1. Introduction

There are two billion overweight or obese individuals worldwide, and more than 25% of these are over 18 years old [1]. Overweight and obese were defined as excessive accumulation of fat, which may cause chronic diseases including diabetes, cardiovascular disease, and cancer. The most useful criterion is body mass index (BMI, kg/m^2^), which is calculated by dividing body weight (kg) by the square of height (m^2^). Overweight is defined as a BMI greater than or equal to 25 kg/m^2^ and obesity as a BMI greater than or equal to 30 kg/m^2^ [1]. Obesity in this population rose from about 4.5% in 1975 to about 13% in 2016, while the number of those overweight increased from about 22% to about 39% [1]. Accumulation of fat is a crucial factor that increases morbidity and mortality [2,3]. Overweight and obese individuals have a higher rate of chronic diseases [4,5]. A previous meta-analysis reported that being overweight and obese is associated with a higher rate of disease-specific and all-cause mortality [6]. Decreasing accumulation of fat is a preventive health behavior that includes maintaining a normal weight, normal BMI, low visceral fat, and increasing lean body mass.

The beneficial effects of exercise participation were keeping a normal weight, low BMI, and low visceral fat [7]. A short-bout exercise (less than 30 min) in a previous meta-analysis with eighteen studies decreased body mass index (BMI), body fat percentage, skinfold thickness, and fat mass [8]. Also, as a result of exercise participation, increased lean body mass in overweight and obese individuals helped increase basal metabolic rate, which can prevent the accumulation of fat [9]. Increased regular physical activity that leads to a negative energy balance helps reduce body weight and fat mass in overweight and obese individuals due to increased lipid oxidation [10]. However, the effects of current exercise interventions are conflicting. A previous meta-analysis study reported a beneficial effect of high-intensity interval training on visceral fat in overweight and obese individuals [11], but those interventions included exercise and diet. Also, a randomized controlled trial found preventive effects on fat-free mass during weight loss when overweight and obese older adults underwent a high-protein diet plus resistance exercise intervention consisting of 1 h sessions 3 times per week for 10 weeks [12]. Another meta-analysis study found reduced weight, BMI, and visceral fat, and increased lean body mass after completing exercise and diet interventions. Lifestyle interventions that included exercise and diet in a previous meta-analysis study also showed decreased accumulated ectopic fat, which is the deposition of triglycerides within non-adipose tissue, including liver, heart, pancreas, and intramyocellular lipids [13]. Although a previous meta-analysis of exercise interventions reported increased adiponectin, which maintains body homeostasis and decreases atherogenesis, diabetes, and inflammation [14], the participants were obese children and adolescents [15]. As opposed to combined exercise and diet interventions, exercise-only interventions may help to better understand and develop exercise interventions, and overweight and obesity in children and adolescents is different than in adults and older adults. Thus, it is important to investigate the effects of exercise-only interventions on the body weight, BMI, visceral fat, and lean body mass of overweight and obese individuals, including adults and older adults. Therefore, the purpose of this meta-analysis was to understand the effects of exercise interventions on overweight and obese individuals on body weight, BMI, visceral fat, and lean body mass. Our meta-analysis was limited to exercise because isolating exercise effectiveness in overweight and obese individuals leads to specific exercise prescription guidelines. It also shows overweight and obese individuals meaningful reasons for the need to participate in exercise to maintain normal body weight and fat mass. This approach also brings to light any limitations associated with exercise-only interventions in overweight and obese individuals, suggesting the combination of exercise interventions with different diets and caloric restriction.

## 2. Materials and Methods

### 2.1. Article Search Process

The Preferred Reporting Items for Systematic Reviews and Meta-Analysis statements (PRISMA) [16] guided our analysis and the MEDLINE and EMBASE databases were used to identify relevant studies dated January 1990 to July 2019. Search terms for eligible articles were accumulated fat, obesity (overweight, adipose tissues), and exercise (aerobic, endurance, strength, resistance). Two researchers (J.L. and H.L.) searched for relevant studies, independently, based on the inclusion and exclusion criteria. If any disagreements arose, further discussions were conducted to reach agreements. The inclusion criteria were reporting results of the effects of pre- and post-exercise interventions, recruiting overweight or obese individuals aged more than 18 years old, being a randomized controlled trial, indicating determination methods for being overweight and obese, and describing measurement technologies including computed tomography (CT), magnetic resonance imaging (MRI), and dual-energy X-ray absorptiometry (DAX). The exclusion criteria were being a pilot study, systematic review, or meta-analysis review, and reporting combined interventions such as diet and exercise. We also manually searched references cited in review articles to identify further relevant studies. The Cochrane Collaboration’s Risk of Bias Tool was used to assess the quality of the selected studies [17]. Quality assessments were performed by two researchers (J.L. and H.L.) individually and the final assessment results were reported. The risks of bias assessment evaluated seven domains that included random sequence generation, allocation concealment, blinding of participants and personnel, blinding of outcome assessment, incomplete outcome data, selective reporting, and other bias. The quality assessment indicates whether the selected studies comply with the seven domains or not, instead of presenting a total score of quality.

### 2.2. Statistical Analysis

The Comprehensive Meta-Analysis 2nd version software (Biostat, Englewood, NY, USA) was used to compute effect size. The standardized mean difference statistic, which is the difference between treatment and control group means divided by the pooled standard deviation, was used to calculate the effect size. Heterogeneity among study results was assessed with the Q test. If *p*-values were less than 0.10, we considered the results to be heterogeneous. Inconsistency was determined based on the values of the Higgin’s I^2^ statistic. If the Higgin’s I^2^ statistic was <50%, it was considered a small inconsistency, and if it was ≥50%, then a large inconsistency. In the heterogeneous case, we used a random effects model, and in the homogeneous case, we used a fixed effects model. Publication bias across studies was assessed by visual inspection of the funnel plot, the rank correlation proposed by Begg et al. [18], and the linear regression proposed by Egger et al. [19].

## 3. Results

**Figure 1:**
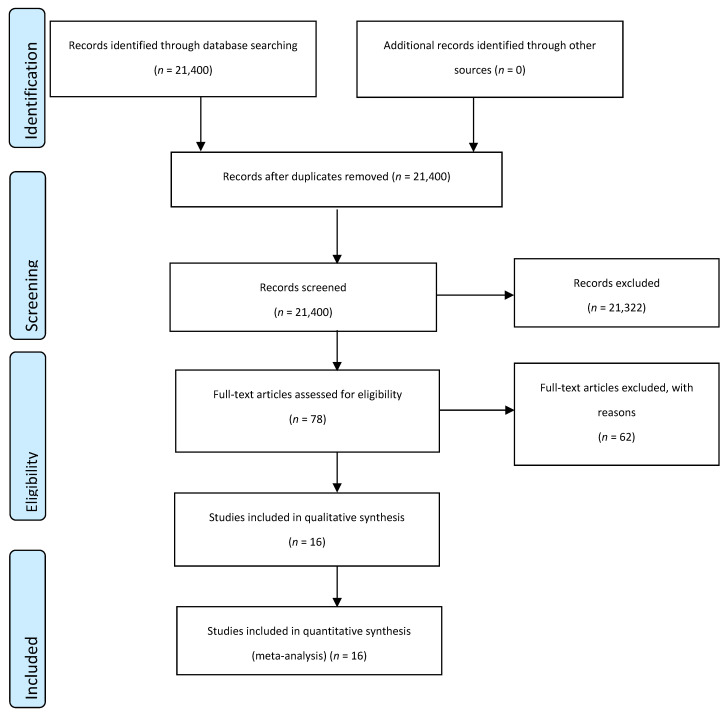
Selection process for the systematic review and meta-analysis.

The article selection processes are presented in Figure 1. The article selection process in our study was based on the PRISMA guidelines. These guidelines exclude unpublished studies and articles not written in English. The search strategy was revised by including a figure of the PRISMA selection process. Figure 1 shows the PRISMA guidelines. The initial search found a total of 21,400 studies, and 21,322 of these studies were excluded due to not being related to our topic of exercise interventions and accumulated abdominal fat. The full texts of 78 studies were reviewed and 62 of those were excluded due to a lack of pre- and post-exercise intervention outcome measures and/or not being a randomized controlled trial. Finally, a total of 16 studies were included in the meta-analysis [20,21,22,23,24,25,26,27,28,29,30,31,32,33,34,35].

**Figure A1:**
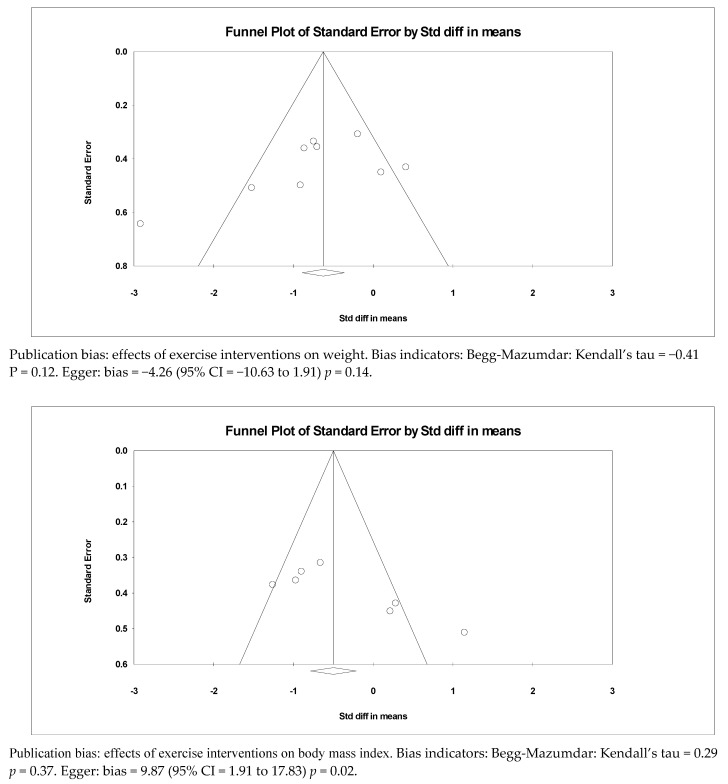

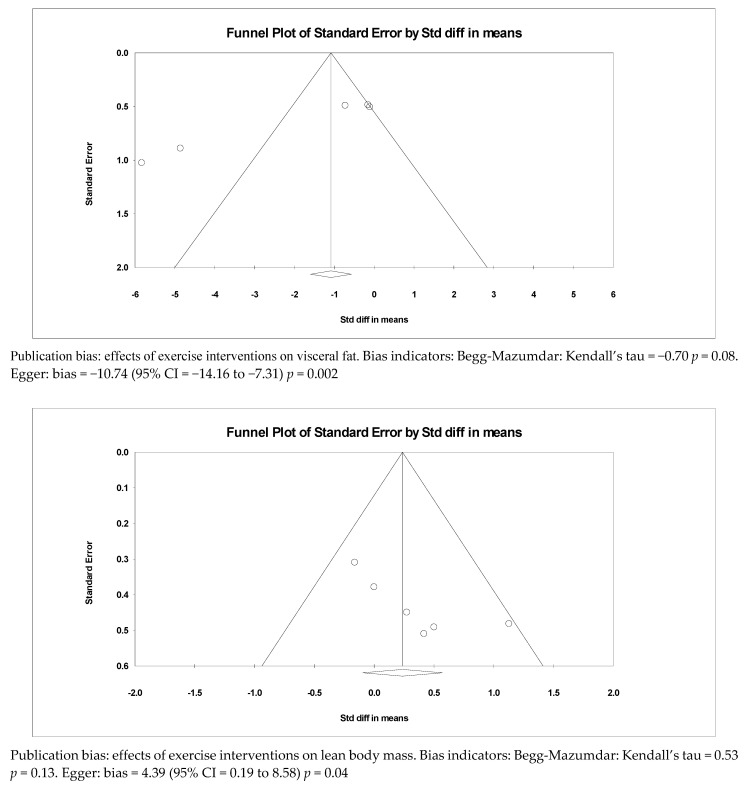
Publication bias.

The basic characteristics of the selected studies including the first author’s name, design of the study, number of participants, levels of BMI, sex, content of exercise interventions, and major findings are presented in Table 1. All participants had to be older than 18 years. The exercise type in 12 of the selected studies was aerobic, 3 studies used resistance exercise, 1 study used interval exercise, and 5 studies used combined exercise that included aerobic exercise such as jogging, walking, and cycling, plus resistance exercise. The average participation in the exercise interventions was 22 weeks, 4 times per week for ~50 min. The average number of participants for each exercise intervention was 35. The average intensity of exercise was from moderate to vigorous. The quality assessment is presented in Table 2. While the assessment tool did not give cutoff scores, we found low bias in the selected studies when evaluated among the seven domains. The effect size was calculated if at least two studies reported the same outcome measures. The effect size of each outcome was calculated if the selected studies provided pre- and post-outcome measures with mean and standard deviation (SD) in both experimental and control groups. While a total of 16 studies were selected, only 5 studies presented a complete set of those outcome measures to enable the proper calculation of effect size. The results of the funnel plot are shown in Appendix A (Figure A1). Visual inspection of the funnel plot revealed some asymmetry. A significant publication bias for weight was detected, but the publication bias existed in BMI, visceral fat, and lean body mass as well.

### 3.1. Effects of Exercise Interventions on Body Weight

**Figure 2:**
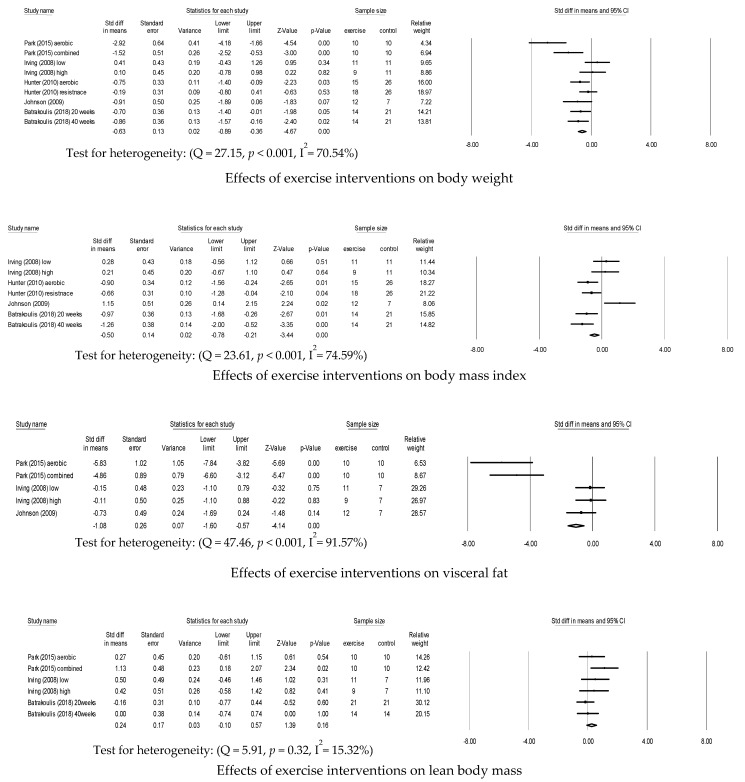
Effects of exercise interventions on overweight and obese individuals.

Nine trials reported weight changes after completing exercise interventions in Figure 2. Overweight and obese individuals who participated in exercise interventions had significantly decreased weight (*d* = −0.63 (95% confidence interval (CI), −0.89–−0.36; *p* < 0.001; *k* = 9)) compared to overweight and obese individuals who did not participate in the exercise interventions.

### 3.2. Effects of Exercise Interventions on BMI

BMI was measured in seven trials (Figure 2). Participants engaging in exercise interventions had a significantly decreased BMI (*d* = −0.50 (95% CI, −0.78–−0.21; *p* < 0.001; *k* = 7)) compared to participants who did not engage in the exercise interventions.

### 3.3. Effects of Exercise Interventions on Visceral Fat

Five trials reported the effects of the exercise interventions on visceral fat in Figure 2. The exercise interventions were effective in reducing visceral fat (*d* = −1.08 (95% CI, −1.60–−0.57; *p* < 0.001; *k* = 5)) in overweight and obese individuals.

### 3.4. Effects of Exercise Interventions on Lean Body Mass

The lean body mass of the participants of the exercise interventions was measured in six trials (Figure 2). While lean body mass increased in these participants compared to those not participating in the exercise interventions, the change was not statistically significant (*d* = 0.26 (95% CI, −0.11–0.63; *p* = 0.17; *k* = 6)).

## 4. Discussion

The exercise interventions reduced body weight, BMI, and visceral fat and increased lean body mass in overweight and obese individuals, but the effects on lean body mass were not statistically significant. Further studies are needed to confirm these findings because of the limited number of studies used in this meta-analysis. The average exercise intervention necessary to elicit beneficial effects was of moderate to vigorous intensity, 50 min a day, 4 times per week, and of aerobic type.

### 4.1. Effects on Body Weight, BMI, Body Fat Percentage, and Waist Circumference

Our meta-analysis found beneficial effects on body weight, BMI, and visceral fat. A previous meta-analysis of three studies reported that overweight or obese individuals benefited from exercise interventions in terms of reducing body weight, BMI, body fat percentage, and waist circumference, and increasing lean body mass [36]. Another meta-analysis also reported reduced visceral fat, but the exercise interventions only included high-intensity interval training [11] and our major exercise type was aerobic. Not surprisingly, exercise intervention type and duration influenced the results. Our meta-analysis did not allow for subgroup analyses to compute effect size by exercise type, intensity, or duration due to the limited number of studies used. On the other hand, we found high heterogeneity of effect size for weight, BMI, and visceral fat. Further studies examining exercise type, intensity, and duration are, therefore, warranted. Furthermore, while the exercise interventions were effective in reducing visceral fat in overweight or obese individuals, the degree of effectiveness varied according to the method of measurement (either CT or MRI). Considering the differences in measurement methods, further studies are needed to delineate clinically meaningful measurements of accumulated fat.

### 4.2. Effects on Lean Body Mass

While lean body mass was increased in overweight and obese individuals who participated in exercise interventions, it was not statistically significant. A previous study reported that increased protein intake during their exercise interventions reduced lean body mass loss in older adults [37]. Another study showed that abdominally obese women participating in endurance and endurance strength interventions increased total lean body mass and physical capacity and decreased fat mass, waist and hip circumferences, resting heart rate, and systolic and diastolic blood pressures [38]. The subgroup used to analyze the effects on lean body mass in our meta-analysis included one combined aerobic and resistance exercise trial and five aerobic only exercise trials. Exercise interventions with additional aerobic exercise or resistance exercise protocols and other exercise types including combined exercise and interval training must be the focus of future investigations to properly conduct the effect size analysis by exercise type.

### 4.3. Understanding of Exercise Interventions

The average exercise intervention in our meta-analysis consisted of moderate to vigorous intensity, duration of 50 min per session, and frequency of 4 times per week for 22 weeks. The exercise modalities included aerobic, resistance, combined aerobic and resistance, and interval training, but the prevalent exercise type was aerobic. These details are important when designing an evidence-based exercise intervention for overweight or obese individuals. The exercise intensity and duration in our results were similar to the recommendations of the American College of Sports Medicine (ACSM) for long-term weight loss. The ACSM recommends 200–300 min per week of moderate intensity exercise. A previous meta-analysis reported that the highest reduction in visceral fat was affected by aerobic exercise of moderate to vigorous intensity [39]. The length of the exercise interventions in our selected studies was on average 22 weeks and ranged between 4 and 48 weeks. Maintaining weight loss and preventing weight regain after completion of the exercise interventions need to also be addressed in future studies through long-term follow-up periods.

### 4.4. Potential Mechanisms of Exercise Effects for Oveweright and Obese Individuals

There are four potential mechanisms that could explain the findings in our study. First, exercise helps increase skeletal muscle metabolism which increases muscle mass and strength, skeletal muscle glucose uptake, and fatty acid oxidation [40]. Second, the effects of exercise on liver tissue may include increased hepatic uptake of fatty acids and decreased hepatic glucose production, cholesterol synthesis, and glycogen synthesis [41]. Third, in adipose tissue, exercise works to reduce fat mass and leptin and resistin production and increase lipolysis and adiponectin production [42]. Last, participating in exercise decreases chronic inflammation and increases growth factor production, leading to endocrine changes that improve systemic mechanisms [43].

### 4.5. Limitations of Our Meta-Analysis Study

This study has several limitations. First, the number of selected studies was too limited for generalizing our findings to all overweight or obese individuals. Additional studies need to be conducted. Second, the studies in our meta-analysis did not control for daily activity and dietary intake in either control or exercise groups. This influenced the weight, BMI, visceral fat, and lean body mass outcomes. Controlling for daily activity and dietary intake may help to better understand the effects of exercise interventions for overweight and obese individuals and weed out confounding variables. Third, our suggested guidelines for exercise interventions in overweight or obese individuals are based on the interventions described in the studies analyzed, but the guidelines require further details. Fourth, our suggested exercise interventions are derived from the average interventions in the selected studies that may elicit different effects on overweight and obese individuals. Fifth, the subjects included in this meta-analysis were either adults or older adults and, therefore, additional studies need to consider other age groups to refine our recommendations. Caution is necessary when applying these guidelines to younger populations and individuals who are not overweight or obese. Sixth, unpublished studies were excluded because they may influence our results; therefore, further studies are needed. Seventh, the results of publication bias may be due to the small number of qualifying studies analyzed. A greater sample size is needed for additional meta-analyses that might include unpublished studies. Last, this meta-analysis included all races. New exercise guidelines should be developed while taking into consideration racial differences.

## 5. Conclusions

Overweight or obese individuals who participated in exercise interventions achieved reduced body weight, BMI, and visceral fat compared to individuals who did not participate in the exercise interventions. Our meta-analysis also found increased lean body mass, but it was not statistically significant. Recommendations of exercise in overweight and obese individuals include all types of exercise, including aerobic and resistance exercise, moderate to vigorous intensity exercise programs, 50 min 4 times per week, and a 22-week duration. These recommendations may be the most effective based on the available evidence. Further studies are needed to confirm the effects of these exercise interventions in overweight and obese individuals depending on exercise type, intensity, duration, and individual measurements of fatness.

## Figures and Tables

**Table 1:** Exercise intervention characteristics of selected studies.

First Author (Year)	Design/Numbers, Body Mass Index (BMI), and Age of Participants	Exercise Intervention	Major Outcomes
Batrakoulis (2018) [20]	Randomized Control Trials (RCT): control (n = 21), exercise (n = 14), vs. exercise-detraining (n = 14), Overweight or obese (25.1–34.9 kg/m^2^), average 36 years old	40 weeks, 3 times/week, combined exercises including aerobic exercise, resistance exercise, and neuromotor exercise, moderate intensity	A whole-body dual-energy X-ray absorptiometry scanner
Besnier (2015) [21]	RCT: moderate intensity (n = 46), resistance training (n = 46), 60% aerobic exercise (n = 45), vs. home exercise (n = 45), Obese older adults (27–40 kg/m^2^), average 20–40 years old	moderate intensity (cycle-ergometers, 60% maximal amount of oxygen uptake (VO_2_max), 4 days/week, 55 min)	Dual X-ray absorptiometry (DXA)
Blue (2018) [22]	RCT: short interval training (n = 18), long interval training (n = 16), vs. control (n = 9), Obese adults (25–45 kg/m^2^), 18–50 years old	8 weeks, short interval training (10 repetition of 1 min bouts, 90% peak power output), long interval training (5 repetition of 2-min bouts, 80–100% peak power output)	Muscle cross-sectional area and thigh fat thickness (ultrasound), lean mass and fat mass of legs (DXA)
Coker (2009) [23]	RCT: moderate-intensity exercise (n = 6), high-intensity exercise (n = 6), vs. control (n = 6), Overweight or obese (26 ≤ BMI < 37 kg/m^2^), 65–90 years old	12 weeks, 1000 kcal energy expend: cycle-ergometers, 50% of VO_2peak_, or 75% of VO_2peak_	Fat mass and lean tissue: X-ray, abdominal subcutaneous adipose tissues and abdominal muscle wall: computed tomography (CT)
Gepner (2018) [24]	RCT: exercise (n = 139), vs. control (n = 139) Overweight and obese older adults (27–41 kg/m^2^), ≥55 years old	18 months, 60 min, 65% MHR of aerobic training, 80% of MHR of resistance training (2 sets, leg extension, leg curl, elbow flexion, triceps extension, lateral pull-down, lower back extension, bent leg sit-ups)	Visceral adipose tissue, intrahepatic fat, pancreatic fat, intrapericardial fat, superficial subcutaneous adipose tissue, deep subcutaneous adipose tissue, renal sinus fat, and femur intermuscular adipose tissue (MRI)
Goodpaster (2010) [25]	RCT: physical activity (n = 67), vs. control (n = 63) Obesity (>30 kg/m^2^), 30–55 years old	12 months, moderate intensity physical activity, brisk walking, 60 min, 5 days/week, 10,000 steps/day	Abdominal adipose tissues and hepatic fat contents (CT)
Hunter (2010) [26]	RCT: control (n = 26), aerobic exercise (n = 15), vs. resistance exercise (n = 18), Overweight women (27 ≤ BMI ≤ 30 kg/m^2^), 21–46 years old	1 year, aerobic exercise (week 1: 20 min, 67% maximum heart rate, and then continues duration and intensity increased, week 8: 80 min, 80% of maximum heart rate), resistance exercise (squats, leg extension, leg curl, elbow flexion, triceps extension, lateral pull-down, bench press, military press, lower back extension, and bent leg sit-ups, 10 repetitions and 80% of 1RM)	Whole body lean and fat tissue (X-ray), intra-abdominal adipose tissue, deep subcutaneous adipose tissue, subcutaneous adipose tissue (CT)
Irving (2008) [27]	RCT: control (n = 7), low-intensity exercise (n = 11), vs. high-intensity exercise (n = 9), Obese women, average 51 years old	16 weeks, walking/running, low intensity RPE ~10–12, week 1–2 (300 kcal, 1–2 days/week), week 3–4 (350 kcal, 4 days/week), week 5–16 (400 kcal, 5–6 days/week), high-intensity RPE ~15–17	Body fat, fat-free mass, fat mass, abdominal fat, subcutaneous fat, abdominal visceral fat, mid-thigh fat area, mid-thigh skeletal muscle: CT
Irwin (2003) [28]	RCT: aerobic and resistance exercise (n = 87), vs. control (stretching, n = 86), Overweight or obese postmenopausal women (≥35 kg/m^2^), 50–75 years old	7 weeks, aerobic exercise (60–75% MHR, 45 min), resistance exercise (10 repetitions/2 sets, leg extension, leg curls, leg press, chest press, and seated dumbbell row)	Total body fat, intra-abdominal fat, subcutaneous abdominal fat (CT)
Johnson (2009) [29]	RCT: control (n = 8), vs. exercise (n = 12), Obesity (≥35 kg/m^2^)	4 weeks, a supervised, progressive aerobic exercise, cycle ergometer, total 30–34 min (15 min sessions and 5 min rest), 3 times/week, 50% VO_2peak_ for week 1, 60% for week 2, and 70% for weeks 3 and 4, 15 min sessions and 5 min rest	Hepatic triglyceride concentration and vastus lateralis intramyocellular triglyceride concentration (point-resolved spectroscopy), subcutaneous adipose tissues area, hepatic lipid saturation index (HMRS), visceral adipose tissue area (MRI)
Ko (2016) [30]	RCT: combined exercise (n = 59), vs. control (n = 21), Obese old adults, 60–80 years old	6 months, aerobic exercise (treadmill, 5 days/week, 60–70% VO_2peak_, 30 min, resistance exercise (3 days/week, chest press, shoulder raise, shoulder flexion, leg extension, biceps curl, abdominal crunches, modified push-ups)	Total, abdominal, abdominal subcutaneous, and visceral adipose tissue (MRI) above the L4–5 intervertebral space
Park (2003) [31]	RCT: aerobic training group (n = 10), combined training group (n = 10), vs. control (n = 10), Overweight or obese (25–35 kg/m^2^), average 40 years old	Aerobic training (60–70% HRmax, 60 min, 6 days/week), combined training groups (3 days/week for resistance exercise, 3 days/week for aerobic exercise)	Abdominal visceral fat, subcutaneous fat, and visceral fat (CT)
Park (2015) [32]	RCT: combined exercise (n = 10), vs. control (n = 10), Abdominal obese postmenopausal women (≥24 kg/m^2^), average 57 years old	12 weeks, resistance exercise (70% of 1 RM, 10–12 repetitions, 3 days/week, 30 min), aerobic exercise (40–75% HRR, 40 min, 3 days/week)	Visceral fat (CT)
Quist (2018) [33]	RCT: aerobic exercise (n = 21), leisure exercise (n = 21), vigorous exercise (n = 33), vs. control (n = 16), Obese adults (25–35 kg/m^2^), 20–45 years old	6 months, bike exercise (320 kcal/day for women, 42 kcal/day for men), leisure-time exercise of moderate (50–70% VO_2peak_), vigorous intensity (50–70% VO_2peak_)	Body composition (DXA)
Schmitz (2007) [34]	RCT: strength training (n = 71 at year 1, n = 70 at year 2). vs. control (n = 67 at year 1, n = 63 at year 3)¸Overweight or obese (25–35 kg/m^2^), 25–44 years old	16 weeks, 2 days/week, 3 sets of 8–10 repetitions, quadriceps, hamstring, gluteal, pectoral, erector spinae, latissimus dorsi, rhomboid, deltoid, biceps, and triceps muscles	Body composition: DAX, abdominal fat areas (total, subcutaneous, and intraabdominal): CT at the L2–3 interspace
Slentz (2005) [35]	RCT: high amount/vigorous intensity (n = 42), low amount/vigorous intensity (n = 46), low amount/moderate intensity (n = 40), vs. control (n = 47), Overweight and obese (25 ≤ BMI ≤ 35 kg/m^2^), 40–65 years old	8 months, (1) high amount/vigorous intensity (jogging 20 miles/week), (2) low amount/vigorous intensity (jogging 12 miles/week), and (3) low amount/moderate intensity (walking 12 miles/week)	Visceral fat (CT), Subcutaneous fat, Total abdominal fat, body weight

**Table 2:** Assessments of the Cochrane Collaboration’s Risk of Bias.

First Author (Year)	Random Sequence Generation	Allocation Concealment	Blinding of Participants and Personnel	Blinding of Outcome Assessment	Incomplete Outcome Data	Selective Reporting	Other Bias
Batrakoulis (2018) [20]	+	?	?	?	+	?	+
Besnier (2015) [21]	+	+	?	?	+	?	+
Blue (2018) [22]	+	+	+	?	+	+	+
Coker (2009) [23]	+	?	?	?	+	?	+
Gepner (2018) [24]	+	+	?	?	+	+	+
Goodpaster (2010) [25]	+	?	?	?	+	?	+
Hunter (2010) [26]	+	+	?	+	+	?	+
Irving (2008) [27]	+	+	?	?	+	?	+
Irwin (2003) [28]	+	+	+	?	+	+	+
Johnson (2009) [29]	+	?	?	?	+	?	+
Ko (2016) [30]	+	+	?	?	+	+	+
Park (2003) [31]	+	?	?	?	+	?	+
Park (2015) [32]	+	+	?	?	+	?	+
Quist (2018) [33]	+	+	+	?	+	+	+
Schmitz (2007) [34]	+	?	?	?	+	?	+
Slentz (2005) [35]	+	+	?	?	+	+	+

+ = Low risk of bias, ? = Unclear risk of bias.
